# Survey data of Entrepreneurial intention and perceived social support from academics-scientists at Chilean Universities

**DOI:** 10.1016/j.dib.2022.108537

**Published:** 2022-08-12

**Authors:** Eduardo Acuña-Duran, Juan Carlos Oyanedel, Daniela Pradenas-Wilson

**Affiliations:** a Facultad de Economía y Negocios, Universidad Andres Bello, Santiago 7591538, Chile; b Facultad de Educación y Ciencias Sociales, Universidad Andres Bello, Santiago 7591538, Chile; c Facultad de Psicología y Psicopedagogía, Pontificia Universidad Católica Argentina, Buenos Aires C1107, Argentina

**Keywords:** Entrepreneurship, Entrepreneurial intention, Academic entrepreneurship, Innovation, Social support, Planned behavior theory, University, Latin America

## Abstract

The data comes from a survey applied to 1027 scientists working in Chile. The survey was originally distributed to 5571 scientists. The research was carried out with the purpose of explaining the Entrepreneurial intention of the scientists. The survey consisted of 35 items in total, structured in five sections: (a) academic background and previous entrepreneurship (14 items), (b) Entrepreneurial intention (6 items), (c) Attitude towards entrepreneurship (5 items), (d) subjective social perception of support towards Entrepreneurship (3 items) and Control of Perceived Behavior towards Entrepreneurship (6 items). The data are linked to the theoretical model proposed in Acuña_Duran, Oyanedel y Pradenas-Wilson (2021).

**Specifications Table**

**Table:** 

Subject	Entrepreneurship
Specific subject area	Academic Entrepreneurship
Type of data	Microsoft Excel file (.xlsx) Figures (MPlus)
How the data were acquired	Survey data. Collected via SurveyMonkey The sampling framework for this study was composed of all the scientists obtaining a FONDECYT (Chilean National Science and Technology Fund) grant between 2007 and 2017. The data for the construction of the sampling framework was publicly available at the website of FONDECYT, where the names and institutions of the grant winners are published. The sampling framework was composed of 5685 individual academics whose emails were collected via their institutional websites
Data format	Raw
Description of data collection	The sampling framework was 5685 researchers responsible for basic and applied scientific research projects of the national fund for scientific and technological development (FONDECYT) in Chile. With SurveyMonkey, the questionnaire was distributed to those teachers and 1027 respondents completed the survey. The data was anonymized throughout the process.
Data source location	Various universities in Chile (39 universities of Chile) National level
Data accessibility	Repository name: Mendeley Data Data identification number: doi: 10.17632/fn3xz4csn4.3 Direct URL to data: https://data.mendeley.com/datasets/fn3xz4csn4/3
Related research article	Acuña-Duran, E., Pradenas-Wilson, D., Oyanedel, J.C. & Jalón-Gardella, R. (2021). Entrepreneurial intention and perceived social support from academics-scientists at Chilean Universities. Frontiers in Psychology, 12, 2575. doi: 110.3389/fpsyg.2021.682632.

## Value of the Data

• The data represents an important contribution to the empirical study of the Entrepreneurial intention of scientists in Spanish-speaking countries.
• The data represents one of the largest samples in the scientific Entrepreneurial intent study area.
• The data offer the possibility of validating the scales in other populations of Spanish-speaking scientists.
• The data can be of use of researchers and policy makers interested on measuring Entrepreneurial intention in academic settings.
• The data can be replicated for comparative purposes.

## 1 Data Description

The data comes from a questionnaire designed to measure the Entrepreneurial intention of scientists in Chile. The Excel spreadsheet BD_DatainBrief_EI2.xlsx. version 3 available through the online repository, contains raw data for the 34 items. The first 20 correspond to the Likert scales of intention, attitude, perceived subjective social norm and perceived behavioral control. The remaining 14 correspond to questions of general characterization of the population under study [1]. Each of these variables included in the database are described in the "Questions - IE - Backgrounds" document available through the online repository. This same document also details the applied questionnaire.

## 2 Experimental Design, Materials and Methods

The survey, implemented through SurveyMonkey, was distributed by email to 5685 scientists working in Chile. The survey was sent to the whole original dataset. A total of 114 emails bounced, reducing the total sample to 5571. A total of 1040 surveys were responded (18.6%). 13 cases were taken out because the main scales were not fully answered, giving a valid total of 1027 cases fully answered (18.4%).

The applied questionnaire is the validated and adapted Spanish version proposed in the original article [2], based on a English questionnaire focused on Entrepreneurial intention [3]. The survey was administered between the 15 of June and the 16 of July of 2017. The data was collected based on a fully defined online questionnaire based on Likert-type scales, with scores from ranging 1 to 7 (scale mean=4).

For the 1027 cases included in the study, no missing data was found in the responses related to the intention model constructs. Missing data on the background information was recorded as such. No imputation procedures were implemented.

## Ethics Statements

For the construction of the dataset, all subjects were asked for their informed consent. The research was carried our following the procedures outlined by the Declaration of Helsinki and the questionnaire and design comply with the personal data protection requirements established in the Chilean Law 19.628, of 1999, as required by the Doctoral Program in Management Sciences of the University of Santiago de Chile.

## CRediT authorship contribution statement

**Eduardo Acuña-Duran:** Conceptualization, Methodology, Software, Writing – original draft. **Juan Carlos Oyanedel:** Data curation, Writing – review & editing. **Daniela Pradenas-Wilson:** Writing – review & editing.

## Declaration of Competing Interest

The authors declare that they have no known competing financial interests or personal relationships that could have appeared to influence the work reported in this paper.

## Data Availability

Entrepreneurial intention of Chilean Scientists (Original data) (Mendeley Data).
